# A novel copper precursor for electron beam induced deposition

**DOI:** 10.3762/bjnano.9.113

**Published:** 2018-04-18

**Authors:** Caspar Haverkamp, George Sarau, Mikhail N Polyakov, Ivo Utke, Marcos V Puydinger dos Santos, Silke Christiansen, Katja Höflich

**Affiliations:** 1 Helmholtz-Zentrum Berlin für Materialien und Energie GmbH, Hahn-Meitner-Platz 1, 14109 Berlin, Germany; 2 Max Planck Institute for the Science of Light, Staudtstr. 2, 91058 Erlangen, Germany; 3 Institute of Optics, Information and Photonics, Friedrich-Alexander-Universität Erlangen-Nürnberg (FAU), Staudtstr. 7/B2, 91058 Erlangen, Germany; 4 Empa—Swiss Federal Laboratories for Materials Science and Technology, Laboratory for Mechanics of Materials and Nanostructures, Feuerwerkerstrasse 39, CH-3602 Thun, Switzerland; 5 Institute of Physics Gleb Wataghin, University of Campinas, Rua Sergio Buarque de Holanda 777, Cidade Universitaria, 13083-859 Campinas-SP, Brazil; 6 Physics Department, Freie Universität Berlin, Arnimallee 14, 14195 Berlin, Germany

**Keywords:** copper, Cu(tbaoac)_2_, focused electron beam induced deposition, nanostructures, optical properties

## Abstract

A fluorine free copper precursor, Cu(tbaoac)_2_ with the chemical sum formula CuC_16_O_6_H_26_ is introduced for focused electron beam induced deposition (FEBID). FEBID with 15 keV and 7 nA results in deposits with an atomic composition of Cu:O:C of approximately 1:1:2. Transmission electron microscopy proved that pure copper nanocrystals with sizes of up to around 15 nm were dispersed inside the carbonaceous matrix. Raman investigations revealed a high degree of amorphization of the carbonaceous matrix and showed hints for partial copper oxidation taking place selectively on the surfaces of the deposits. Optical transmission/reflection measurements of deposited pads showed a dielectric behavior of the material in the optical spectral range. The general behavior of the permittivity could be described by applying the Maxwell–Garnett mixing model to amorphous carbon and copper. The dielectric function measured from deposited pads was used to simulate the optical response of tip arrays fabricated out of the same precursor and showed good agreement with measurements. This paves the way for future plasmonic applications with copper-FEBID.

## Introduction

The focused electron beam in a scanning electron microscope can be used to deposit material. This process is often observed as an unwanted side effect in electron microscopy. Residual chamber gases, adsorbed on the substrate surface, are decomposed by the electron beam and become visible as a darkening of the irradiated area [1]. By introducing a volatile precursor gas into the vacuum chamber [2–3] this focused electron beam induced deposition (FEBID) enables the fabrication of three-dimensional structures with nanometer precision [4]. The respective precursor gas is locally supplied by a capillary needle. The electrons decompose the adsorbed molecules and leave non-volatile fragments on the substrate while the volatile fragments are pumped out of the chamber [2]. Due to the small spot size of the electron beam in combination with the patterning possibilities of varying point distances and dwell times, three-dimensional shapes with high lateral resolution can be fabricated [4–6]. There is ongoing research for new precursors to improve the quality of the deposits and expand the choice of materials [2,7]. To deposit metallic structures by FEBID, typically a metal-organic precursor is used, which frequently results in a carbonaceous matrix with small metal inclusions [8].

Fabrication of copper-containing deposits by electron beam induced deposition was shown with Cu(I) and Cu(II) precursors containing the ligand hexafluoroacetylacetonate (hfac, C_5_H_1_F_6_O_2_) bound to the copper atom [9–10]. With these precursors, both planar structures and nanopillars were realized. These precursors led to metal contents between 11 atom % [9] and 25 atom % [10] for the as-deposited material. The deposited material from Cu(hfac)_2_ showed an insulating or highly resistive behavior. The resistivity of 30 Ω·cm could be measured after thermal purification only [11–12]. Using a precursor with the additional ligand trimethylvinylsilane (vtms), namely Cu(I)(hfac)(vtms), pure freestanding copper rods were obtained after a post growth purification during electron beam induced heating [13].

All prior investigated copper precursors contain fluorine. Since fluorine is not only toxic but also highly reactive, a fluorine-free precursor is highly desirable for the integration into commercial electron microscopes. A possible standard precursor for copper deposition should furthermore provide for conductive deposits with a preferably high copper content.

Here, the metal-organic precursor bis(*tert*-butylacetoacetato)Cu(II) (CAS: 23670-45-3, C_16_H_26_CuO_6_) is introduced as a fluorine-free alternative. This precursor is known from chemical vapor deposition (CVD), leading to planar copper films with resistivities as low as 2.9 µΩ·cm and copper contents of around 74 atom % [14]. In case of FEBID it shows reliable deposition even for complex three-dimensional geometries and leads to conductive deposits with copper contents around 25 atom %. The deposits were investigated concerning their morphology, composition and electrical properties. Transmission and reflection spectra of planar deposits of various heights served as input for the retrieval of the complex permittivity of the copper-containing material. The obtained values were used to numerically model the spectrum of FEBID nanocones.

## Results and Discussion

**Figure 1:**
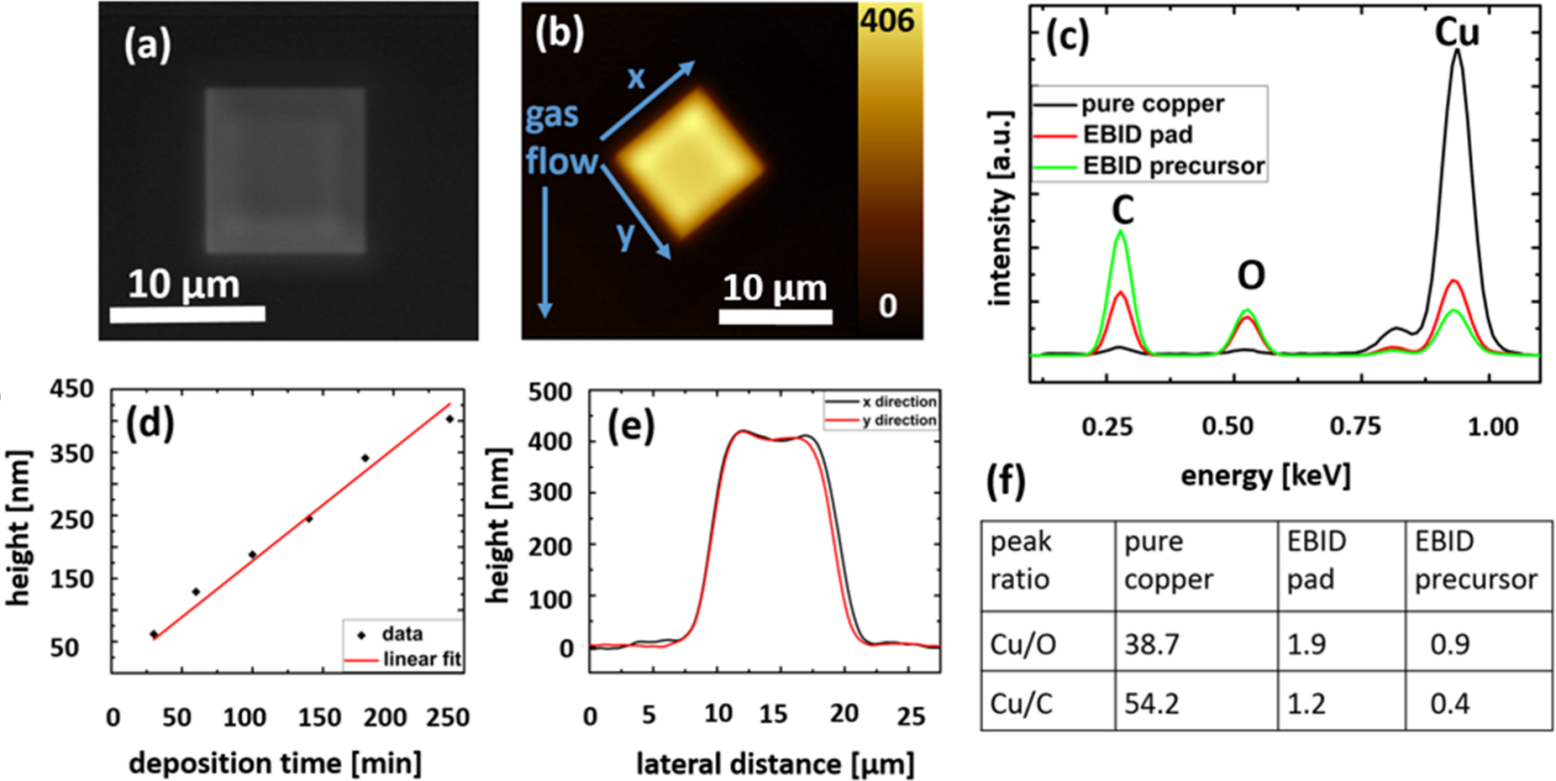
(a) Scanning electron micrograph of a FEBID pad. (b) Atomic force micrograph of the same pad. (c + f) EDX spectra of the solid crystalline precursor, a copper foil with a purity >99.999% and a FEBID pad grown on a silicon substrate with a height above 400 nm. The table shows the peak ratios of copper to oxygen and copper to carbon (d) Height of FEBID pads for different deposition times, written with a dwell time of 10 μs and a point distance of 3 nm. (e) Atomic force microscope line scans of the FEBID pad in (a) and (b).

Pads with different deposition times were fabricated and their heights were measured by atomic force microscopy, cf. Figure 1a and Figure 1b. The deposition current was measured to be 7 nA. Other deposition parameters are 15 keV primary beam energy, 10 μs dwell time, 3 nm point-to-point distance using a serpentine scanning routine. The number of scans increased from 30 to 300.

The square pads had an edge length of 10 μm. The AFM image revealed the top edge of the pad being higher than the point in the center, giving an indented shape. This indicates a precursor-limited growth regime, due to the high current, where the middle of the scanning area became precursor-depleted and thus the deposition rate decreased. For calculating the deposition rate of the novel precursor, the volume of the pads was calculated by the pad area (100 μm^2^) multiplied by their height measured in the middle of the pads. Using this method, the high edges of the pad and the slants of the sides were neglected. The resulting volume deposition rate R = 0.026 µm^3^·nA^−1·^min^−1^ is comparable to the deposition rate of common platinum precursors [15]. However, it has to be pointed out that in our case the deposition rate was precursor-limited.

In Figure 1c the spectra energy-dispersive X-ray spectroscopy (EDX) on the pristine precursor, on the deposits, and on pure copper for reference are shown. The determination of the composition of the precursor was challenging since the precursor already decomposed during the measurement by the impact of the electron beam. Peak ratios of copper to oxygen and copper to carbon are given in Figure 1f for the pristine precursor, the FEBID deposits and pure copper. The stoichiometry of the precursor gives an atomic composition of Cu:O:C of 4 atom %:26 atom %:70 atom %. The EDX measured copper content was 7% above the stoichiometry value, likely due to the simultaneously decomposition under electron beam impact. For EDX on a deposit, the pad thickness was chosen such that the spectrum shows no signal from the silicon substrate, therefore the complete signal originates from the FEBID pad itself. The measurement shows a high copper content of 24 atom %, which is a large increase compared to the pure precursor. The spectrum also indicates that mostly carbon is removed during the deposition process, while the oxygen content stays constant within the reliability of the measurement. A room temperature four-point-probe electrical measurement yielded a conductive material with 1.26 MOhm in the as-deposited state. The estimated resistivity was 1 Ohm·cm, what is six orders of magnitude larger than the value for pure copper [16]. However, the presented precursor shows the first evidence for conductivity of the deposits without post-treatment in case of copper [2,10].

**Figure 2:**
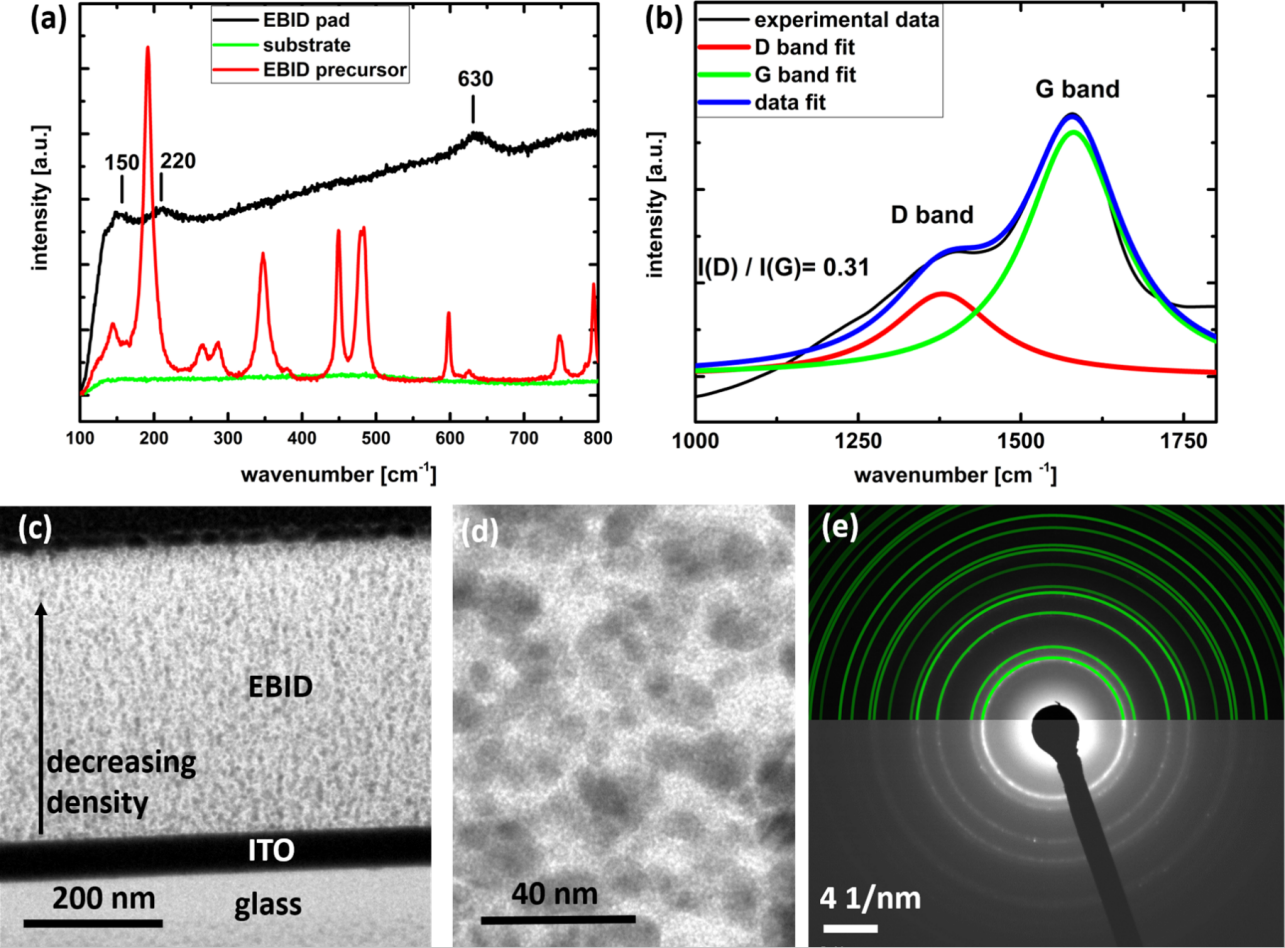
(a) Raman spectra of a FEBID pad, FEBID precursor and the substrate. The complex structure of the precursor leads to a variety of spectral features. Indicated are three Raman lines for Cu_2_O [17] corresponding well with the ones measured in the FEBID pads. (b) Raman features of the amorphous carbon matrix in the FEBID material. (c) Cross-section TEM image of a FEBID pad. (d) High-magnification TEM of FEBID pad. (e) SAED indexing of copper particles in the FEBID material, with all diffraction rings corresponding to indicated green Cu diffraction rings.

For a better understanding of the material configuration and change during deposition, Raman spectroscopy measurements were performed on the precursor before deposition as well as on the FEBID pads. The Raman spectrum in Figure 2a of the precursor shows a complex structure with a number of distinct peaks. Under the impact of the electron beam the spectrum changes to a broad Raman response (black curve in Figure 2a) that is typical for amorphous materials. The different features of the material before and after FEBID suggest a complete break of all bonds present in the crystalline structure. The three small peaks visible in the deposited FEBID pad at 150, 220 and 630 cm^−1^ suggest a partially oxidized copper state of the copper particles [17]. This is important in view of the dielectric function of the resulting material since the permittivity of a metal depends on the oxidation state. To survey the oxidation state in detail, cross-sections of FEBID deposits were investigated by transmission electron microscopy (TEM). Figure 2c and Figure 2d show the inner FEBID structure of the copper deposits, with crystallites below ≈20 nm in diameter. Selected area electron diffraction (SAED) of the deposit (Figure 2e) yields diffraction rings which fully correspond to pure Cu, with no rings corresponding to Cu oxides. SAED was carried out for different regions within the deposit covering a significant part of the available cross-sectional deposit area (cf. Supporting Information File 1). Thus, the peaks of copper oxide in the Raman signal (visible in Figure 2a) most likely originate from copper particles oxidized at the surface of the deposit. This indicates that inside the FEBID material the copper is un-oxidized. Hence, the optical response of the composite is expected to be determined by pure copper particles dispersed in a dielectric carbonaceous matrix.

The regions around 1300 cm^−1^ and 1500 cm^−1^ in the Raman spectrum displayed in Figure 2b provide information about the configuration of this carbon. One larger peak is visible around 1580 cm^−1^, as well as one minor peak around 1350 cm^−1^. These peaks are referred to as D for disordered and G for graphite [18]. The intensity ratio of the D to G peak is a measure of the amorphization state of carbon. The low value of 0.3 for the FEBID carbon, as well as the G-peak position of 1580 cm^−1^, indicates a highly amorphous carbon structure inside the deposit [19].

**Figure 3:**
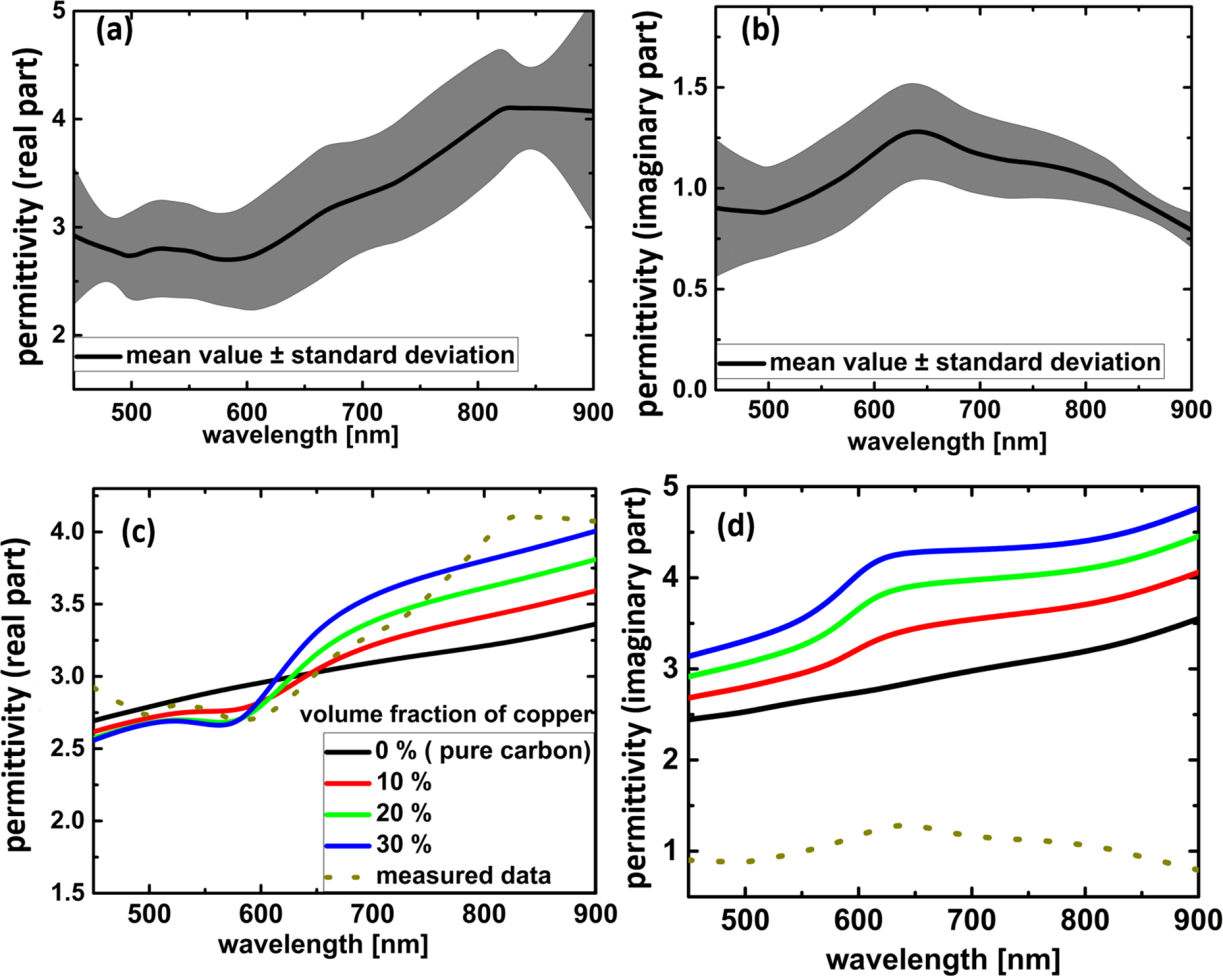
(a) Real and (b) imaginary part of the dielectric function of the measured FEBID material, averaged over 5 pads together with the standard deviation. (c) Real and (d) imaginary part of the Maxwell–Garnett model. The model uses the dielectric function of copper from [24], and of carbon from [23]. The dotted lines are the measured values.

The determination of reliable values for the optical response of FEBID materials is difficult due to the long deposition times for large areas. This makes standard measurements like ellipsometry very time consuming or even unrealistic. To find the permittivity values for the investigated copper precursor, µ-spectroscopy on the deposited pads was used [20]. Reflection and transmission were determined for FEBID pads with side lengths of 10 × 10 μm deposited on a glass substrate covered with a 50 nm layer of ITO. A brute force algorithm compared the obtained spectra to analytically calculated spectra of the corresponding multilayer system by scanning the n-k-space for the FEBID material. Thereby, the optical constants were retrieved [20]. Figure 3a and Figure 3b show the values for the real and imaginary part of the dielectric function averaged over 5 pads. The grey regions indicate the standard deviations to show the large fluctuations observed. A possible explanation is the inhomogeneous distribution of the copper particles, observed in the cross-sectional view of the copper FEBID pads (Figure 2c). In contrast to other FEBID materials [20], no correlation between the pad thickness and the dielectric function was found.

Since the metallic inclusions have sizes far below the wavelength of light, the description of the FEBID material as an effective medium is conceivable. In this case the metal particles are treated quasistatically as dipole scatterers. The analytical formula for dipole scatterers in a dielectric matrix is given by the Maxwell–Garnett (MG) approach. A large uncertainty in modeling the dielectric function of the FEBID composite stems from the unknown optical properties of the carbonaceous matrix. The dielectric behavior of the carbonaceous matrix is most probably influenced by the large portion of oxygen and even by some contained hydrogen. Both are expected to optically dilute the material [21–22]. In addition, carbon occurs in multiple configurations with very different properties. For the carbon matrix, the Raman spectrum showed a highly amorphous carbon phase. Hence, modified values for amorphous carbon from Hagemann et al. [23] are used. In view of the results obtained from SAED, the particles are described using the Johnson and Christie values for copper [24]. Figure 3c and Figure 3d show the results from the MG theory of FEBID-copper for three different copper concentrations. The retrieved permittivity is added as a dotted line for the real and imaginary part. While the modeled real part matches the measurements, the measured imaginary part shows lower values than the model. This suggests that the permittivity values assumed for the carbon phase are too high. A possible reason is the high oxygen content. Measurements on hydrogenated carbon with different oxygen contents have shown that the refractive index decreases significantly with increasing oxygen content [21–22].

**Figure 4:**
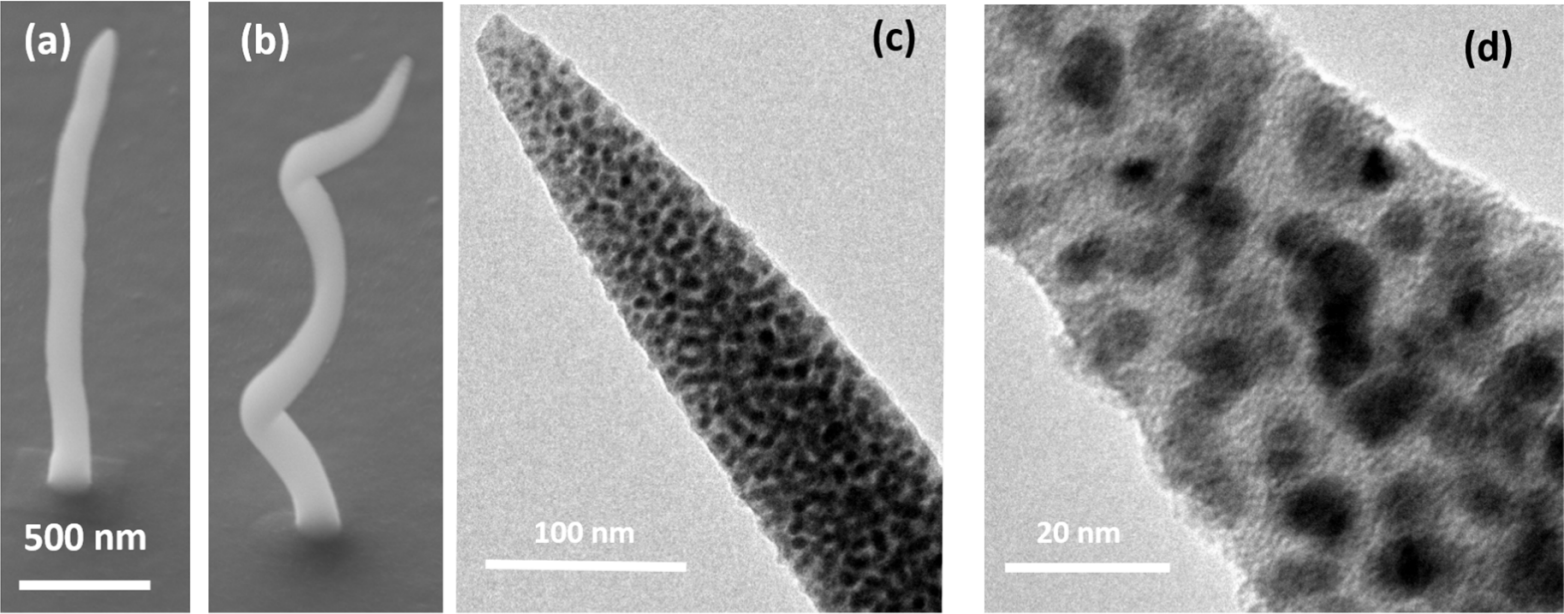
(a + b) Scanning electron micrographs of a single nanopillar (a) and a copper helix with three pitches (b) deposited on glass covered with 50 nm ITO, the scale of (a) and (b) is the same. (c + d) Transmission electron micrographs of a copper pillar. Visible is the typical FEBID structure of copper particles embedded in an amorphous matrix.

Ideally, the retrieved value can now be employed for the optical description of nanostructures. Nanostructures were fabricated using an acceleration voltage of 15 kV with strongly reduced beam currents around 200 pA to achieve an optimal resolution. Needles were deposited using 50 pA beam current and point irradiation times of 60 seconds. The helix was achieved by a circular pattern with a radius of 120 nm, point-to-point distance of 0.5 nm and dwell time of 30 ms using two repetitions for two helix turns. Figure 4 shows scanning electron micrographs of both types of nanostructures (a) needle, (b) helix. The TEM images in Figure 4c and Figure 4d show the typical structure of a FEBID material. The dark dots are copper particles embedded in an amorphous carbon matrix, which appears light. From the high-resolution TEM, the particles can be estimated to have diameters around 10 nm, resembling well the structure of the 2D deposit. The average EDX signal from nanopillars gives a composition of 26 atom % Cu, 13 atom % O and 61 atom % C.

**Figure 5:**
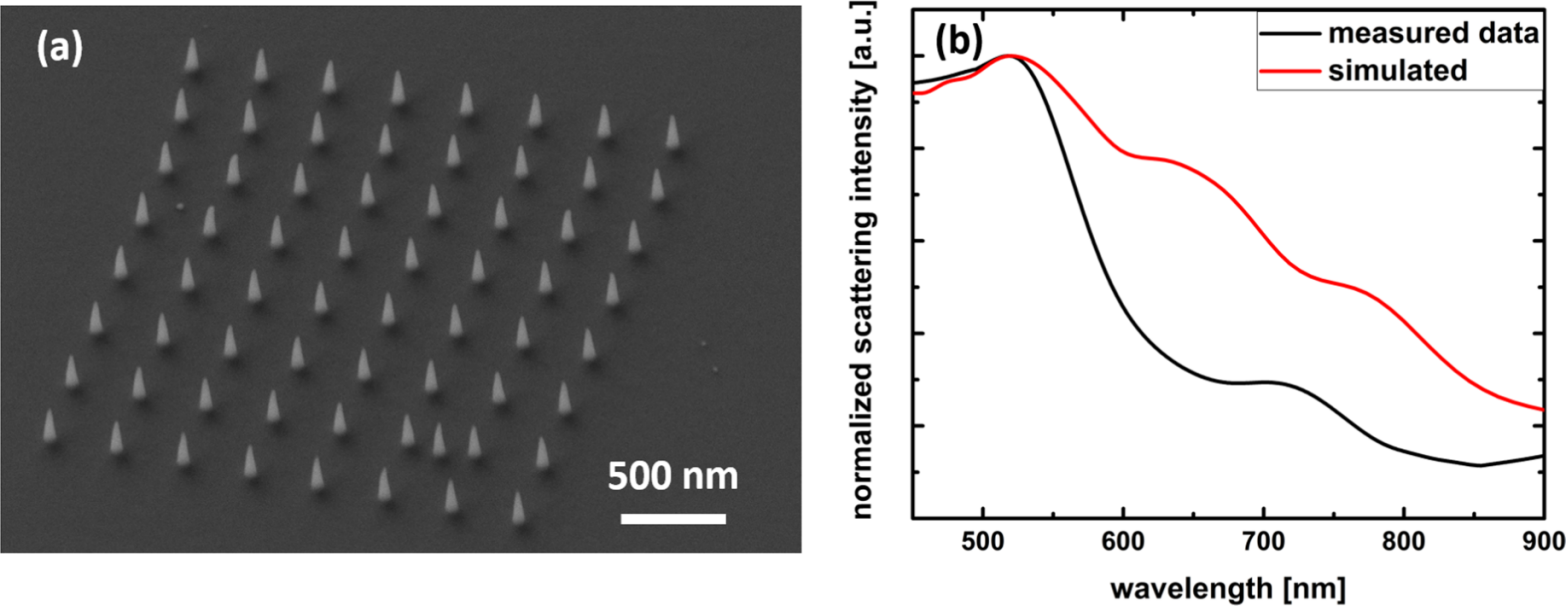
(a) Array of 8 × 8 nanocones with a distance of 400 nm, base diameter of 80 nm and a height of 250 nm. (b) Measured and simulated scattering spectra of the array.

Hence, the retrieved mean value for the refractive index may serve as a meaningful estimation to simulate the optical properties of an array of 8 × 8 nanocones with a distance of 400 nm and a base diameter of 80 nm (Figure 5a). It was fabricated using 50 pA beam current and a dwell time of 8 seconds for each cone. The scattering intensity was measured by dark-field reflection spectroscopy and compared to FDTD simulations. Figure 5b shows the measured and simulated scattering intensities. The as-deposited structures exhibit a resonance around 550 nm. The simulation with the retrieved permittivity resembles the resonance of the as-deposited cones.

## Conclusion

A novel fluorine-free copper precursor was introduced for FEBID. The precursor showed good deposition properties at substrate and GIS temperatures of around 100 °C. Complex three-dimensional structures like helices could be realized. EDX analysis showed a relatively high metal content of around 24 atom % copper when compared to other metal-organic copper precursors for FEBID. The copper nanoparticles were embedded in an amorphous carbon and oxygen containing matrix. Raman investigations proved a high degree of carbon amorphization. TEM observations revealed the diffraction pattern of pure copper inside the deposits, while the Raman signal indicates the presence of copper oxide on the deposit surface, probably due to post-deposition oxidation. The room temperature resistivity was about 1 Ohm·cm showing Ohmic behavior. The permittivity of the material in the visible spectral range was determined by reflection/transmission measurements and a brute force algorithm, based on a transfer matrix method. The material showed a dielectric behavior for all investigated pad heights. The general behavior of the material could be described by the Maxwell–Garnett mixing model with the permittivity’s of amorphous carbon and copper and their respective volume fractions as input parameters. In conclusion, this study presents a promising novel copper precursor compound for focused electron beam induced deposition which is well-suited for direct writing of three-dimensional device parts.

## Experimental

The deposition experiments were carried out in a Tescan electron microscope MIRA, equipped with a gas injection system designed by modular flow [25] and assembled by Kammrath and Weiß.

The GIS reservoir was manually filled before each deposition. The reservoir and needle were separately heated to 100 °C for the reservoir and to 105 °C for the needle. The GIS needle opening was adjusted approximately 1 mm above the sample surface with a total working distance of 16 mm. The stage was heated to 100 °C. The chamber pressure during the deposition was around 5 × 10^−4^ mbar. The chamber pressure increases to 5 × 10^−2^ mbar the first time the valve to the precursor chamber is opened at 100 °C. This is most likely due to the release of water absorbed by the crystal precursor. After the normal pressure is restored, opening and closing the valve does not lead to any measurable pressure change in the vacuum chamber, suggesting a vapor pressure below 5 × 10^−4^ mbar. As substrates n-doped silicon wafer pieces with a native oxide layer and glass cover slips with an optically characterized layer of 50 nm indium tin oxide (ITO) were used. EDX measurements were carried out in a Tescan LYRA 3 dual beam microscope equipped with an EDX Quantax system of Bruker. Spectra were taking in spot mode at an accelerating voltage of 5 kV and 1 nA beam current. To avoid spurious signals from the substrate a deposit of 400 nm thickness onto silicon was used for quantification. Further EDX measurements were carried out on the optically characterized copper deposits on ITO coated glass (see Supporting Information File 1). In this case EDX was performed using a Hitachi S 4800 equipped with an EDAX silicon drift detector using an acceleration voltage of 8 kV and 1 nA beam current. The obtained k-ratios were evaluated using the software Stratagem according to a routine described earlier [26] and provided copper contents which were consistent with the substrate-free measurement.

The optical spectra were taken with a Zeiss Axio Imager optical microscope. All samples were illuminated with unpolarized light of a halogen lamp through a 100× objective with a numerical aperture of 0.75. The light is collected with an objective and out-coupled through a 400 μm optical fiber to a Horiba iHR 320 spectrometer. By use of the fiber, the spectrometer collects light from a 1 μm spot.

The Raman measurements were carried out in a micro-Raman setup in a backscattered configuration using a LabRam HR800 (Horiba Scientific). The light source is a linearly polarized laser, emitting at a wavelength of 457 nm. A 100× objective lens (numerical aperture 0.9) is used to focus the laser beam onto the sample, resulting in a spot size of about 700 nm. The spectra were taken with an Horiba iHR 320 spectrometer.

Transmission electron microscopy (TEM) images onto the nanostructures were acquired with a Gatan Orius CCD-Camera inside a CM12 (Phillips) at an accelerating voltage of 120 kV equipped with a EDAX Genesis silicon drift detector. For TEM investigations the nanostructures were directly deposited onto Omniprobe molybdenum TEM grids. Cross-sectional samples from planar deposits for imaging by TEM were prepared by a focused ion beam (FIB) lift-out technique in a Zeiss Crossbeam 340 KMAT. TEM on the cross-sections was performed on a JEOL JEM2200fs CM12. SAED pattern indexing was carried out using CSpot software (CrystOrient).

Electrical measurements were performed at room temperature using a conventional four-probe setup with a Keithley 2400 source meter. The power dissipation on the deposits was limited to 1 nW to avoid self-heating and changes in atomic composition. The current voltage curve is provided in Supporting Information File 1.

## Supporting Information

File 1 Additional information on EDX measurements, SAED indexing, and electrical characterization.
