# Supplementary material for: A novel copper precursor for electron beam induced deposition
Source: Beilstein J Nanotechnol. 2018 Apr 18;9:1220–7. doi: 10.3762/bjnano.9.113 (PMC5942376; doi:10.3762/bjnano.9.113)
Supplement: File 1 — Additional information on EDX measurements, SAED indexing, and electrical characterization. [file Beilstein_J_Nanotechnol-09-1220-s001.pdf]

Supporting Information

for

A novel copper precursor for electron beam induced deposition

Caspar Haverkamp[1,2], George Sarau[2,3], Mikhail N. Polyakov[4], Ivo Utke[4], Marcos V. Puydinger dos Santos[4,5], Silke Christiansen[1,2,6] and Katja Höflich*[1,2,4]

Address: [1]Helmholtz-Zentrum Berlin für Materialien und Energie GmbH, Hahn-Meitner-Platz 1, 14109 Berlin, Germany, [2]Max Planck Institute for the Science of Light, Staudtstr. 2, 91058 Erlangen, Germany, [3]Institute of Optics, Information and Photonics, Friedrich-Alexander-Universität Erlangen-Nürnberg (FAU), Staudtstr. 7/B2, 91058 Erlangen, Germany, [4]Empa–Swiss Federal Laboratories for Materials Science and Technology, Laboratory for Mechanics of Materials and Nanostructures, Feuerwerkerstrasse 39, CH-3602 Thun, Switzerland, [5]Institute of Physics Gleb Wataghin, University of Campinas, Rua Sergio Buarque de Holanda 777, Cidade Universitaria, 13083-859 Campinas-SP, Brazil and [6]Physics Department, Freie Universität Berlin, Arnimallee 14, 14195 Berlin, Germany

*Corresponding author

Email: Katja Höflich - katja.hoeflich@helmholtz-berlin.de

Additional information on EDX measurements, SAED indexing, and electrical characterization

EDX Measurements

Standardless EDX quantification relies on several assumptions like equal distribution of elements within the bulk (no microstructure, no layers), known absorption cross-sections and a well-characterized detector in a system of stable contamination[1]. The used software implementation is property of the respective manufacturer. Hence, this type of quantification suffers from relatively large errors and the comparability between different measurement systems is hampered.

All types of deposits were characterized by EDX. The main manuscript presents EDX results of a deposit of a sufficient thickness to avoid signal intensity from the substrate. These EDX measurements were carried out in a Tescan LYRA 3 dual beam microscope equipped with an EDX Quantax system of Bruker. Spectra were taking in spot mode at an accelerating voltage of 5 kV and 1 nA beam current.

In case of the thin planar deposits on ITO coated glass the EDX was performed using a Hitachi S 4800 equipped with an EDAX silicon drift detector using an acceleration voltage of 8 kV and 1 nA beam current. We had to switch the measurement system to be able to extract spectra and k-ratios as raw data files. The obtained k-ratios were evaluated using the software Stratagem according to a routine described earlier[2] and provided copper contents which were consistent with the substrate-free measurement. The thick deposits on the samples were used to compare the quantification of the different measurement systems, since they showed only a minor signal from the substrate. Table T1 shows the corresponding evaluation for one of the samples. The heights of the deposits were determined using AFM.

	C	O	Cu	Thickness (nm)	Density ($g.cm^{-3}$)
**PAD 1.1**	57.5%	16.9%	25.7%	407.8	2.43
**PAD 1.2**	68.4%	4.4%	27.3%	182.8	2.11
**PAD 2.1**	56.3%	17.2%	26.5%	442.7	2.50
**PAD 2.2**	58.6%	14.8%	26.6%	340.9	2.40
**PAD 2.3**	60.6%	13.1%	26.3%	245.2	2.45
**PAD 2.4**	66.4%	7.0%	26.6%	187.5	2.18
**PAD 2.5**	72.0%	1.9%	26.1%	129.8	2.24
**PAD 3.1**	76.0%	0.0%	24.0%	62.7	3.15

Table T1: Results of EDX quantification for deposits onto ITO-coated glass with deposition parameters as given in the main manuscript. After extraction of the the k-ratios the software Stratagem was used for thin-film correction.

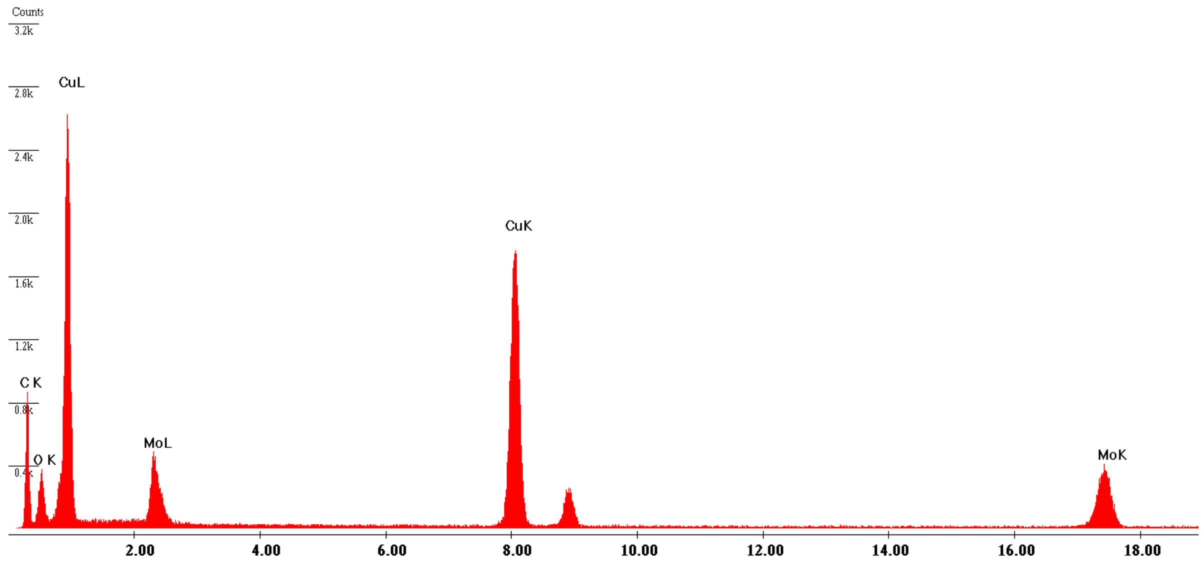

Figure S1: EDX spectrum of a copper nanopillar taken in the TEM using an Edax silicon drift detector in spot mode at 120 kV.

The nanopillars were characterized by EDX inside the TEM. One of the obtained spectra for an acquisition time of 100s in spot mode is displayed in Figure S1. To ensure a quantification that is as reliable as possible, molybdenum TEM grids were used as the substrate for deposition instead of the standard copper grids. As stated in the manuscript, the quantification yielded a copper content of 26 at.% using the software of the manufacturer.

SAED indexing

Cross-sectional samples from planar deposits for imaging by TEM were prepared by a Focused Ion Beam (FIB) liftout technique in a Zeiss Crossbeam 340 KMAT. TEM on the cross-sections was performed on a JEOL JEM2200fs CM12. Selected area electron diffraction (SAED) pattern indexing was carried out using CSpot software (CrystOrient). Figure S2 (a) displays an overview image with the regions of indexing indicated by numbers. The aperture size displayed in Figure S2 (b) is ~600 nm diameter, so each SAED pattern is from almost the entire deposit thickness.
To the best of our knowledge, SAED is the most suitable way to determine crystal structure. While HRTEM can give an indication of lattice spacings and crystal structure, and EELS yields information relating to composition and bonding states, SAED is the most sensitive way to determine crystal structure in the TEM. With the SAED patterns and by using reference Pt SAED patterns, we can definitively say that the crystalline portions of the depositions are in an FCC Cu configuration, and the crystallites do not match either Cu_2O or CuO. This is illustrated in Figure S2 (c), where all of the diffraction rings from the entire deposit thickness matched.

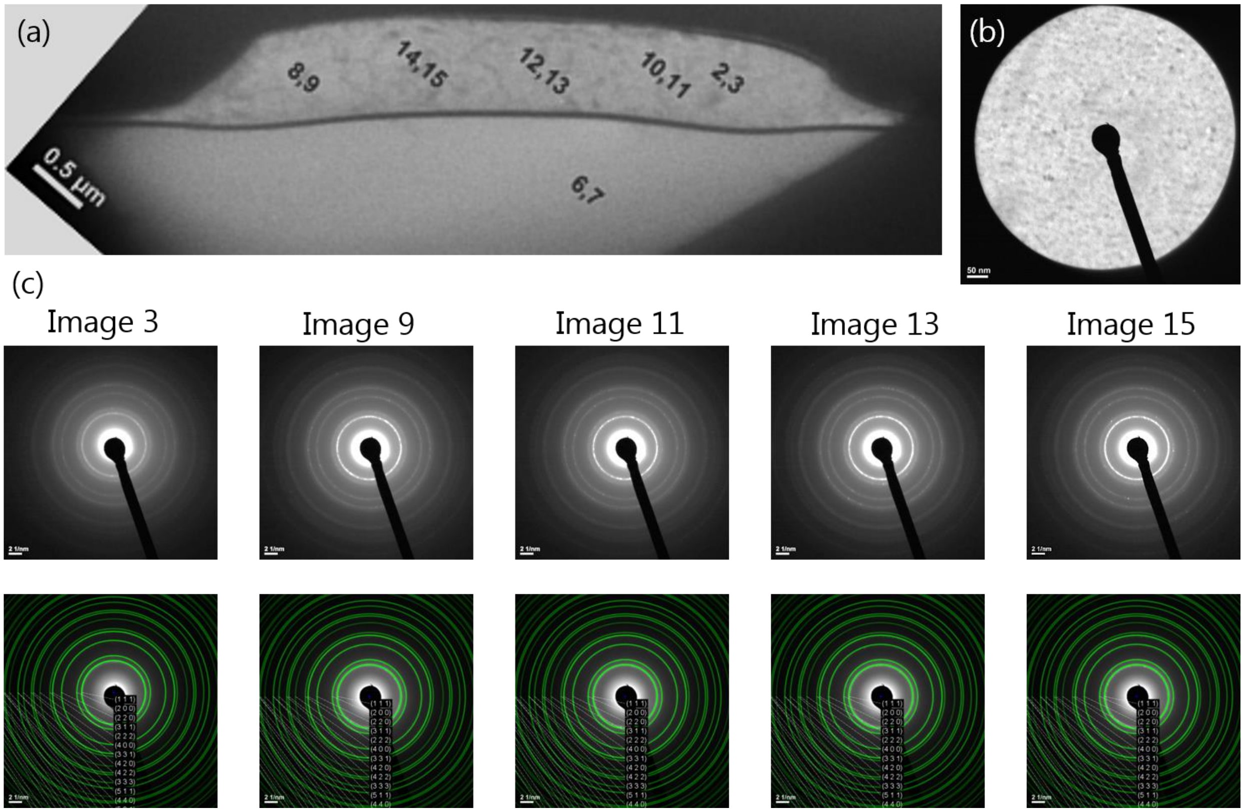

Figure S2: Results of SAED indexing onto copper deposit lamella.

It is possible that there is a minor fraction of Cu in the amorphous portion of the deposit that is bonded to O or some other element, and perhaps EELS could reveal that in a future study. However, since O is not soluble in FCC Cu, we can say that the crystalline FCC Cu is pure and not bonded to oxygen.

Electrical Characterization

Electrical measurements were performed at room temperature using a conventional four-probe setup with a Keithley 2400 source meter. The power dissipation on the deposits was limited to 1 nW to avoid self-heating and changes in atomic composition. The sample was prepared by sputtering of gold electrodes onto a 200 nm silicon oxide on silicon substrate. Figure S3 (a) shows a scanning electron micrograph of the sample layout together with the copper deposit. The obtained current voltage curve with Ohmic behavior and the calculated resistance of ~1 MΩ are displayed in Figure S3 (b).

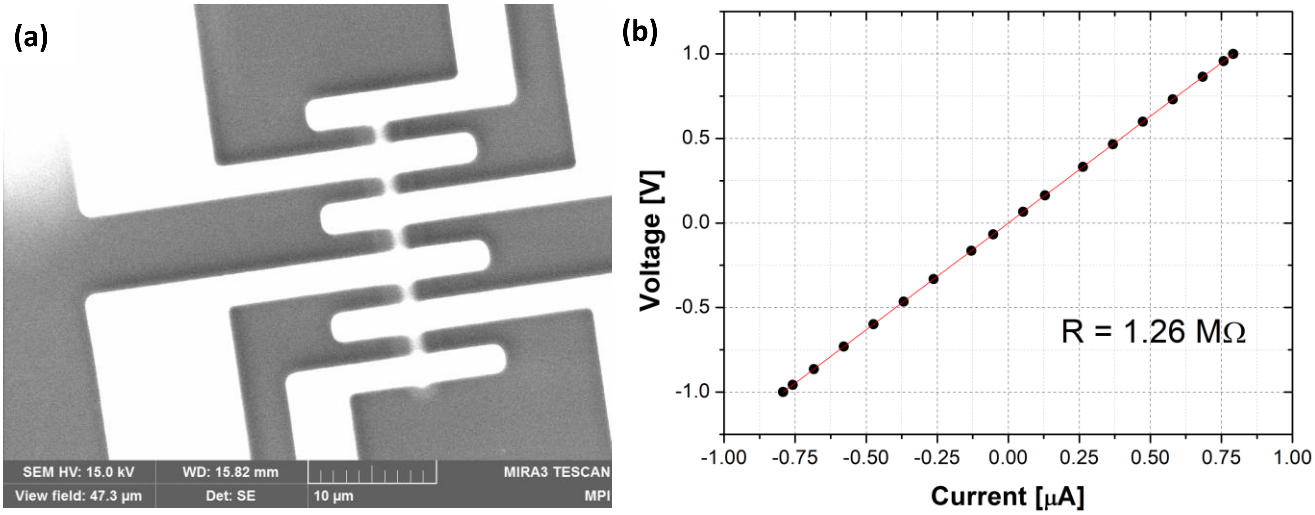

Figure S3: (a) Scanning electron micrograph of sample layout with copper deposit directly after fabrication. (b) Current voltage curve for a planar copper deposit obtained from four point probe characterization. The red line shows the linear fit.

References

(1) Newbury, D. E.; Ritchie, N. W. M. *J. Mater. Sci.* **2014**, *50* (2), 493–518.

(2) Höflich, K.; Jurczyk, J.; Puydinger, M.; Zhang, Y.; Kapusta, C.; Utke, I.; Puydinger dos Santos, M. V.; Götz, M.; Guerra-Nuñez, C.; Best, J. P.; Kapusta, C.; Utke, I. *ACS Appl. Mater. Interfaces* **2017**, *9* (28), 24071–24077.
